# Recombinant Vaccinia Viruses Coding Transgenes of Apoptosis-Inducing Proteins Enhance Apoptosis But Not Immunogenicity of Infected Tumor Cells

**DOI:** 10.1155/2017/3620510

**Published:** 2017-08-30

**Authors:** Olga Koval, Galina Kochneva, Anastasiya Tkachenko, Olga Troitskaya, Galina Sivolobova, Antonina Grazhdantseva, Anna Nushtaeva, Elena Kuligina, Vladimir Richter

**Affiliations:** ^1^Department of Biotechnology, Institute of Chemical Biology and Fundamental Medicine, SB RAS, Novosibirsk, Russia; ^2^Department of Natural Sciences, Novosibirsk State University, Novosibirsk, Russia; ^3^Department of Viral Hepatitis, State Research Center of Virology and Biotechnology “Vector”, Rospotrebnadzor, Koltsovo, Russia

## Abstract

Genetic modifications of the oncolytic vaccinia virus (VV) improve selective tumor cell infection and death, as well as activation of antitumor immunity. We have engineered a double recombinant VV, coding human GM-CSF, and apoptosis-inducing protein apoptin (VV-GMCSF-Apo) for comparing with the earlier constructed double recombinant VV-GMCSF-Lact, coding another apoptosis-inducing protein, lactaptin, which activated different cell death pathways than apoptin. We showed that both these recombinant VVs more considerably activated a set of critical apoptosis markers in infected cells than the recombinant VV coding GM-CSF alone (VV-GMCSF-dGF): these were phosphatidylserine externalization, caspase-3 and caspase-7 activation, DNA fragmentation, and upregulation of proapoptotic protein BAX. However, only VV-GMCSF-Lact efficiently decreased the mitochondrial membrane potential of infected cancer cells. Investigating immunogenic cell death markers in cancer cells infected with recombinant VVs, we demonstrated that all tested recombinant VVs were efficient in calreticulin and HSP70 externalization, decrease of cellular HMGB1, and ATP secretion. The comparison of antitumor activity against advanced MDA-MB-231 tumor revealed that both recombinants VV-GMCSF-Lact and VV-GMCSF-Apo efficiently delay tumor growth. Our results demonstrate that the composition of GM-CSF and apoptosis-inducing proteins in the VV genome is very efficient tool for specific killing of cancer cells and for activation of antitumor immunity.

## 1. Introduction

Oncolytic viruses are novel multifunctional anticancer agents with increasingly promising outcomes in patients [1]. They can directly lyse tumor cells and be vectors coding specific molecules (proteins or RNAs with regulatory functions), which assist in killing or inhibiting the growth of tumor cells, and stimulate the immune system [2]. Viral proteins interact with a number of intracellular signaling pathways; thus, it is expected that they have the potential to regulate various cell death modalities. These include apoptosis, necrosis, necroptosis, pyroptosis, and autophagic cell death, often with one as the predominant form of death for a particular OV [3]. An overwhelming majority of adenoviruses induces autophagic cell death [4]. The highly attenuated vaccinia virus, GLV-1h68, preferentially downregulates antiapoptotic proteins, resulting in an overall shift in protein expression within the cell, favoring apoptosis, while wild VV causes predominantly programmed necrosis [3, 5–7]. Moreover, it was earlier believed that reovirus induces apoptosis of infected cells, but new molecular classification indicates reovirus-induced cell death as necroptosis in addition to apoptosis [8, 9]. Since OVs usually code many proteins, helping viruses to avoid host immune response, various recombinant OVs with cytokines or other immunostimulatory molecules were constructed for overcoming such immunosuppression [10–12]. Indeed, recombinant VVs that expressed immunostimulatory transgene, for example, GM-CSF or the CD40 ligand, had an advanced therapeutic activity against various tumors [13–15].

Attenuated vaccinia virus has shown great potential as an oncolytic virus acting with safety and some efficacy in preclinical and clinical trials [16]. The large genome of VV can easily accept insertions of foreign genes without substantially compromising viral replication. Moreover, the cytoplasmic localization of virus particles in host cells prevents the interference of virus DNA with cellular DNA. These properties allow various manipulations of the vaccinia genome to construct recombinant VVs with reinforced antitumor action. Recently, several classes of chemotherapeutics have been shown to cause immunogenic cell death (ICD), which is characterized by the release of immunomodulatory molecules that activate antigen-presenting cells and thus trigger the induction of more potent anticancer adaptive immune responses with tumor-specific immune memory development [17, 18]. Preapoptotic exposure of calreticulin (CRT), postapoptotic release of the high-mobility group box 1 protein (HMGB1), adenosine triphosphate (ATP) secretion, and their interaction with phagocytosis receptors are required for ICD and antitumor immunity [19]. Furthermore, there is emerging evidence that certain oncolytic viruses and conventional ICD inducers (chemotherapeutics and UV radiation) activate a similar danger response, leading to anticancer immunity [3, 20–23].

Although the vaccinia virus has been shown to preferably trigger programmed necrosis we reported in our previous investigation that the double recombinant vaccinia virus VV-GMCSF-Lact, coding proapoptotic protein lactaptin and human GM-CSF, induced cancer cell death with caspase-3 and caspase-7 activation [6, 24]. Nevertheless, the effect of VV-GMCSF-Lact on the other checkpoint elements of the apoptotic cascade, as well as the induction of immunogenic cell death, has not been investigated yet. We have also previously engineered a recombinant vaccinia virus VVdGF-ApoS24/2, coding apoptin, and this virus exhibited significantly higher selective lytic activity in human cancer cells than the parental strain L-IVP [25]. Apoptin (or VP3 protein), a 14 kDa nonstructural protein from chicken anemia virus, which specifically kills tumor cells, was chosen as a transgene for construction of other double recombinant VVs [26, 27]. Here, we attempted to understand whether VVs, armed with apoptosis-inducing proteins, shift the death type of infected tumor cells from necrosis to apoptosis. Our additional goal was to test such recombinant VVs for induction of immunogenic death of cancer cells in vitro.

## 2. Materials and Methods

### 2.1. Cells and Animals

African green monkey kidney fibroblasts (CV-1) were obtained from the American Type Culture Collection (ATCC; Manassas, VA). The RLS murine lymphosarcoma cells were generously provided by Dr. V. I. Kaledin (Institute of Cytology and Genetics SB RAS, Novosibirsk, Russia). The MX-7 murine rhabdomyosarcoma and MDA-MB-231 human breast adenocarcinomas were obtained from the Russian cell culture collection (Russian Branch of the ETCS, St. Petersburg, Russia). Cells were maintained as previously published [28].

Female SCID mice (6–8 weeks old, line SHO-PRKDC SCID HR/HR1EW 43375) were obtained from SPF vivarium of the Institute of Cytology and Genetics SB RAS, Novosibirsk, Russia.

### 2.2. Recombinant VACVs

The Lister strain (L-IVP) of VV was obtained from the State Collection of Viral and Rickettsial Disease Agents of the State Research Center of Virology and Biotechnology “Vector”, Koltsovo, Russia. The recombinant vaccinia viruses, VV-GMCSF-dGF and VV-GMCSF-Lact, were constructed from L-IVP and purified as described previously [24]. VVdGF-ApoS24/2 was used as the parental virus for homologous recombination of GM-CSF human cDNA into the structural part of the* tk* locus [25, 28, 29]. The resultant VV-GMCSF-Apo is a double recombinant VV with the GM-CSF transgene in the* tk* locus and with the apoptin transgene in the virus growth factor* (vgf)* deletion. The apoptin expression cassette includes the VV promoter, the synthetic apoptin gene sequence, and the FLAG-encoding sequence. The structure of recombinant VV-GMCSF-Apo was confirmed by sequencing both DNA strains with primers 5′-CAGAATTAATTAGACGAGTTAGACG (TK-flank1 sense) and 5′-TCTCGGTTTCCTCACCCAAT (TK-flank1 as) for the* tk* region, and 5′-GTAAGCAAAGAATATAAGAATGAAGCGGTAATGAT (Up35) and 5′-CGAGCACAATACCGGGAGATGG (Apa-L22) for the* vgf* region. The expression of GM-CSF and apoptin was tested by western blot as described in [24, 25].

### 2.3. Analysis of Virulence of Recombinant VV Variants in Ova (Chicken Embryos)

Ten-day-old chicken embryonating eggs were inoculated into chorioallantoic membranes with VVs in tenfold dilutions [30]. The dilution range was selected so that the highest dose of the virus caused the death of all the embryos and the lowest dose caused the death of no embryos. The initial titer of all VVs measured in vitro by plaque assay on a CV-1 cell monolayer was 1.0 × 10^9^ PFU/mL. The dilution range in our experiments was 10^1^–10^7^ PFU/egg for each VV variant, corresponding to doses of infection 10^7^–10^1^ PFU/egg. Each recombinant VV was diluted in ten replicates (10 embryos). At 48 h after infection, the eggs were opened and the embryo viability was visually determined. The 50% lethal dose value of chicken embryos (LD_50_) was calculated according to the Karber method. The LD_50_ was expressed as the tenfold dilution causing 50% mortality of embryos.

### 2.4. In Vitro Cell Killing Assay

Tumor cells were seeded at 1.5 × 10^4^ cells per well on 96-well plates. The next day, recombinant viruses were added to the cells and incubated for 72 h. During incubation, cells were analyzed by phase-contrast microscopy using ZOE Fluorescent Cell Imager (Bio-Rad) and the final viability was detected by an MTT test, as described earlier [24].

### 2.5. Flow Cytometry

All analyses were performed using FACSCanto II flow cytometer (BD Biosciences, Franklin Lakes, NJ), and the data were analyzed by FACSDiva Software (BD Biosciences). Cells were initially gated (P1) based on forward scatter versus side scatter to exclude small debris, and ten thousand events from this population were collected.

For detection of apoptosis, cells were harvested with trypsin and stained with annexin V-FITC and PI using the BD Pharmingen Apoptosis Detection Kit (BD Bioscience), according to the manufacturer's protocol. Cell populations with the annexin V^−^/PI^−^ phenotype were designed as living cells, annexin V^+^/PI^−^ as apoptotic cells, and annexin V^+^/PI^+^ as secondary necrotic cells. Caspase-3 and caspase-7 activation was detected, as described previously [24]. In short, all detached and adhesive infected cells were incubated with FLICA working solution from Vibrant FAM Caspase-3 and Caspase-7 Assay Kit (ref. V35118, Molecular probes by Life Technologies), according to the manufacturer's protocol.

For detection of the changes in the mitochondrial membrane potential (Δ*ψ*_*m*_), infected cells were incubated with 5 *μ*g/mL JC-1 (5,5′,6,6′-tetrachloro-1,1′,3,3′-tetraethylbenzimidazolcarbocyanine iodide) (Sigma-Aldrich) for 20 min at 37°C and were detached by trypsin and analyzed by flow cytometry. The number of cells with changed Δ*ψ*_*m*_ was expressed as a percentage of cells with a fluorescence emission shift in the FITC channel compared to control nontreated cells.

For exo-CRT or exo-HSP70 detection, cells were incubated with primary anti-CRT rabbit antibodies (1 : 100, Abcam, UK) or anti-HSP70 mouse antibodies (1 : 100, Abcam, UK) diluted in PBS containing 10% FBS and 2% glycine for 1 h at 23°C. Rabbit IgG (Thermo Fisher Scientific, USA) or mouse IgG (R&D, USA) was used as isotype control. Next, cells were washed twice with PBS, followed by a 1 h incubation with Alexa Fluor 594-conjugated chicken anti-rabbit antibodies (1 : 2000, Invitrogen, Belgium) or Alexa Fluor 555-conjugated goat anti-mouse antibodies (1 : 2000, Invitrogen, Belgium).

### 2.6. Fluorescence Microscopy

For microscopic analysis of the mitochondrial membrane changes in MDA-MB-231 or MX-7 cells, cells were seeded (1 × 10^4^) onto 4-well culture slides (BD Falcon, Bedford, MA), treated with recombinant VVs for 36 h, and then 5 *μ*g/mL JC-1 were added for 20 min. Stained (nonfixed) cells were visualized with a fluorescence microscope, Axioskop 2 PLUS (Carl Zeiss, GmbH).

### 2.7. DNA Ladder

The whole cellular DNA was isolated by standard procedure. Briefly, at the indicated time points after VV infection, detached and adhesive cells were collected and combined in PBS, lysed in buffer containing 20 mM Tris (pH 8.1), 10 mM EDTA, and 1% SDS, centrifuged at 12000 ×g for 10 min, and supernatants were treated with a phenol-chloroform-isoamyl alcohol (25 : 24 : 1) extraction mix. After centrifugation (14000 ×g for 15 min), the aqueous phase was treated with RNAase A (5 *μ*g/ml) at 37°C for 1 h, and DNA was precipitated by ethanol with 0.3 M NaAc, pH 5.3. DNA samples were analyzed by 2% agarose gel electrophoresis.

### 2.8. ATP Assay

Extracellular ATP was measured in the conditioned media using ENLITEN ATP Assay System Bioluminescence Detection kit (Promega), based on luciferin-luciferase conversion according to the following reaction:

ATP + D-Luciferin + O_2_ → Oxyluciferin + AMP + PPi + CO_2_ + Light (560 nm). For the measurement of the light intensity, a luminometer CLARIOstar (BMG Labtech) was used.

### 2.9. Western Blot Analysis

Tumor cells infected by recombinant VVs were lysed in buffer: 50 mM Tris (pH 8.0), 5 mM EDTA, 150 mM NaCl containing 0.1% SDS, 1x complete protease inhibitor cocktail (Roche Diagnostics GmbH, Mannheim, Germany), and 1 mM PMSF. Samples (30 *μ*g) were separated by 10% SDS-PAGE and transferred to a Trans-Blot nitrocellulose membrane (Bio-Rad Laboratories) by a wet blotting procedure (100 V, 500 mA, 90 min, 15°C) using “Mighty Small Transphor” (GE healthcare Bio-Science AB, USA). Immunodetection was performed using iBind system (Life Technologies), iBind Cards (Invitrogen, Thermo Fisher Scientific) and antibodies: ANTI-FLAG BioM2 antibody (Sigma-Aldrich, F9291), BAX (1 : 1000, Abcam), cyclin B1 (1 : 60, Sigma-Aldrich), tubulin (1 : 200, Sigma-Aldrich), GAPDH (1 : 100, R&D), and HMGB1 (1 : 6000, Abcam) and using goat anti-mouse HRP-conjugated polyclonal IgG (1 : 200, Abcam) or donkey anti-goat HRP-conjugated IgG (1 : 200, R&D) as secondary antibodies, with Novex ECL HRP chemiluminescent substrate reagent kit (Invitrogen, USA). A C-DiGit blot scanner (Li-COR Bioscience) was used for luminescent detection. Densitometric analysis of the western blot data was performed using the image analysis software Gel-Pro Analyser (Media Cybernetics) version 3.1.

### 2.10. Mouse Experiment

All procedures involving animals were performed in compliance with the protocols and recommendations for proper use and care of laboratory animals (ECC Directive 86/609/EEC). The protocol was approved by the Committee on the Ethics of Animal Experiments of the Administration of the Siberian Branch of the Russian Academy of Science. Female SCID mice were injected subcutaneously with 1 × 10^6^ MDA-MB-231 tumor cells. When tumor nodes reached approximately 200 mm^3^, animals were divided into groups and received two intratumoral injections of recombinant VV (100 *μ*L, 1 × 10^7^ PFU/mouse) or saline in the control group, as specified in Results. After the first injection, tumor volume and mouse weight were measured twice a week.

### 2.11. Statistical Analysis

A Student's *t*-test was used to compare treatment effects in cell experiments. For mouse experiments, the data are expressed as mean ± SE. The Mann–Whitney *U*-test was used for comparison between the two groups. A *p* value of less than 0.05 was considered significant.

## 3. Results

### 3.1. Apoptin-Coding Recombinant Vaccinia Virus

**Figure 1:**
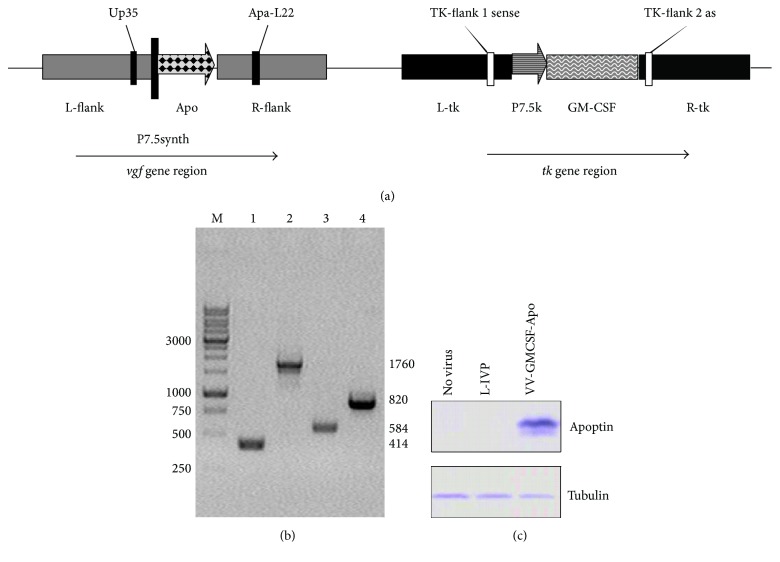
*Verification of recombinant VV-GMCSF-Apo structure and apoptin expression*. (a) Schematic view of VV-GMCSF-Apo genome with primer positions indicated. (b) PCR identification of VV DNA and transgene sequences. Electrophoresis of PCR products with primers TK-flank 1 sense and TK-flank 2 as (Lines 1-2) and with primers Up35 and Apa-L22 (Lines 3-4). Lines 1 and 3: wild-type VV (L-IVP); 2 and 4: VV-GMCSF-Apo. M is DNA molecular weight marker. (c) The expression of apoptin was detected by western blot with monoclonal ANTI-FLAG BioM2 antibody. CV-1 cells were infected with VV-GMCSF-Apo or L-IVP for 24 h.

A new double recombinant vaccinia virus, VV-GMCSF-Apo, bearing GM-CSF and apoptin as transgenes, was constructed to compare the oncolytic action of VVs coding different apoptosis-inducing proteins. The GM-CSF sequence was inserted into the structural part of the viral* thymidine kinase (tk)* gene, and the apoptin sequence was inserted into the* vaccinia growth factor (vgf)* gene deleted region. The apoptin transgene contained a full length sequence of the coding region of the VP3 protein (AF390102, nucleotides 465–830, GenBank) of the chicken anemia virus, and there was no leader sequence for secretion. The engineering scheme of VV-GMCSF-Apo was the same as for the lactaptin-coding VV-GMCSF-Lact described previously [24]. The structure of the recombinant virus was confirmed by both PCR assay with specific primers and DNA sequencing of the* tk* and* vgf* loci (Figure 1(a)). Using primers for the* tk* gene region, the recombinant VV-GMCSF-Apo produced a 1760 bp fragment that corresponds to the* gm-csf* gene insertion, whereas the parental strain L-IVP produced a 414 bp fragment. Using primers for the* vgf* gene region, the fragment size of the recombinant virus with the apoptin gene insertion was 820 bp and 584 bp for the L-IVP strain (Figure 1(b)). Intracellular expression of apoptin in infected CV-1 cells was examined by western blot using commercial antibodies to the FLAG epitope (Figure 1(c)).

To test the attenuation of VV-GMCSF-Apo as well VV-GMCSF-Lact, ten-day-old chicken embryos were infected by various doses of double recombinant VVs and the parental L-IVP virus. LD_50_ was calculated using the ratio of dead and living embryos after infection. We found that the virulence of VV-GMCSF-Apo and VV-GMCSF-Lact was 158- and 100-fold lower than that of the parental L-IVP strain, respectively (Table 1).

### 3.2. Cytotoxicity of Recombinant VVs In Vitro

**Figure 2:**
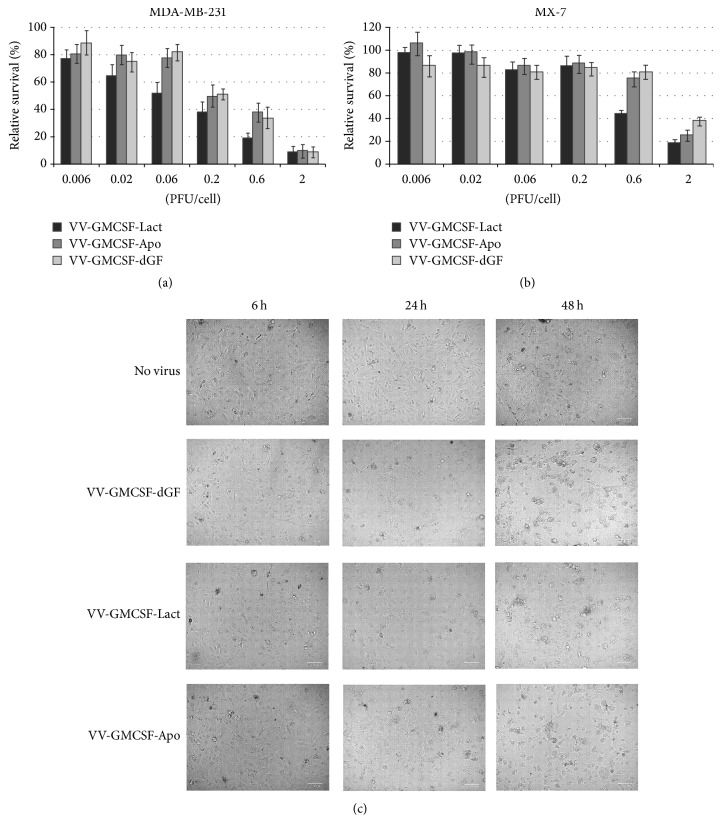
*Cytopathic effects of recombinant vaccinia viruses on tumor cell growth*. (a) MDA-MB-231 and MX-7 cells were infected with various concentration of VVs (0.06–2 PFU/cell) for 72 h. The relative survival of infected tumor cells was detected by MTT test. The data represent the mean ± SD. (b) Visualization of lytic (apoptotic) morphology of infected MDA-MB-231 cells. Cells were infected with VV-GMCSF-dGF, VV-GMCSF-Lact, and VV-GMCSF-Apo. At 6, 24, and 48 h after infection, cells were analyzed by inverted microscope (phase-contrast) and photographed. Representative photomicrographs of three independent experiments.

VV infects a broad range of host cells, killing them with different intensity. To test the cytotoxic activity of recombinant VVs, we used the human cancer cell lines MDA-MB-231 and MCF-7, as well as the mouse cancer cells, methylcholanthrene-induced rhabdomyosarcoma (MX-7) and chemoresistant lymphosarcoma (RLS). The recombinant VV-GMCSF-dGF, containing the GM-CSF transgene in the* tk* gene deletion and additional deletion of the* vgf* gene, was used as a control virus [24]. A dose of 0.6 PFU/cell recombinant VVs caused rapid damage of all the tested cell lines. According to the calculated IC50, the highest cytotoxic activity was detected for VV-GMCSF-Lact (Figure 2(a)). MTT analysis revealed that MX-7 mouse cancer cells were resistant to recombinant VVs, and no recombinant virus induced visible toxic effects against MX-7 cells when multiplicity of infection (MOI) was not up to 0.06 PFU/cell (Figure 2(b)). However, the viability of MDA-MB-231 cells decreased when the MOI of recombinant viruses was 0.006 PFU/cell (Figure 2(a)). The data obtained was confirmed by the phase-contrast microscopy of the cells treated with recombinant VVs (Figure 2(c), see Figures S1 and S2 in Supplementary Material available online at https://doi.org/10.1155/2017/3620510). We observed no monolayer destruction in the MX-7 cells after 48 h of incubation with 0.05 PFU/cell of recombinant viruses, and visual evidence of cell lysis was detected when MOI was increased to 0.5 PFU/cell. In the case of MDA-MB-231 cells, cell death was visually well-detected at 0.05 PFU/cell of recombinant viruses.

### 3.3. Apoptosis Analysis of Infected Cells

**Figure 3:**
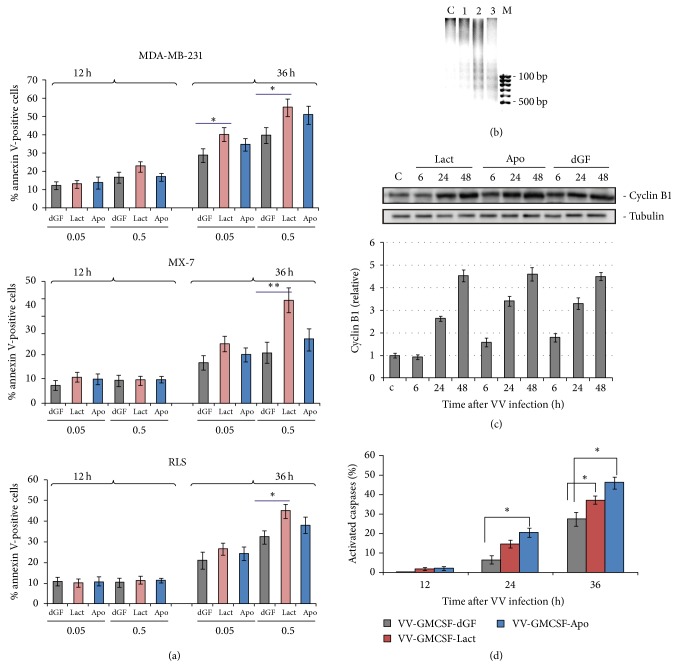
*Apoptosis analysis of infected cells*. (a) Cells were treated with recombinant VVs (0.05 and 0.5 PFu/cell) for 12 and 36 h and then stained using annexin V/propidium iodide (PI). The stained cells were assayed for apoptosis by flow cytometry. Bar graphs summarized the percentage of apoptotic cells from three independent experiments (^*∗*^*p* < 0.05, ^*∗∗*^*p* < 0.01). dGF-VV-GMCSF-dGF, Lact-VV-GMCSF-Lact, and Apo-VV-GMCSF-Apo. (b) Electrophoretic analysis of DNA fragmentation (C: nontreated cells, 1: VV-GMCSF-dGF, 2: VV-GMCSF-Lact, 3: VV-GMCSF-Apo, and M: DNA marker). MDA-MB-231 cells were treated with VVs (0.05 PFU/cell) for 72 h and then DNA samples were isolated. Data are representative of at least two independent experiments. (c) Western blot analysis of cyclin B1 in MDA-MB-231 cells. The quantification of the digital images was performed using Gel-Pro Analyzer software. Data are presented as mean ± SD (*n* = 3). (d) The percentage of the MDA-MB-231 cells with active caspase-3 and caspase-7 in VV-treated cells. Cells were infected with recombinant VVs (0.05 PFU/cell) for 12, 24, and 36 h and then analyzed by flow cytometry as described in Materials and Methods. FAM-positive cells (%) were calculated as the difference between experimental sample and control sample. The data represent the mean ± SD, *n* = 3 independent experiments. ^*∗*^The difference between groups was statistically significant at *p* < 0.05.

The vaccinia virus usually causes lysis of the infected cells, but expression of various transgenes can modulate the host death pathway. To test for apoptosis, we carried out the analysis of annexin V/PI double stained cells infected with recombinant VVs. We observed that, at low MOI as well as at high MOI, the RLS and MX-7 mouse cancer cells were resistant to recombinant VVs after 12 h of infection without visible differences between viruses. After 36 h of infection the RLS and MX-7 cells became sensitive to recombinant viruses, and the largest effect was observed for VV-GMCSF-Lact at high MOI (Figure 3(a)). In contrast to mouse cells, the human cancer cells MDA-MB-231 were sensitive to all three recombinants at low MOI as well as at high virus doses.

DNA fragmentation by endonucleases is a typical late sign of apoptosis. As shown in Figure 3(b), apoptosis was characterized by DNA fragmentation around 72 h after infection. Recombinant VVs coding apoptosis-inducing proteins produced more substantial DNA fragmentation than the control VV-GMCSF-dGF recombinant. Western blot analysis of cyclin B1 in infected MDA-MB-231 cells revealed a strong linear increase of cyclin B1 from 6 to 48 h after infection for all three recombinant viruses (Figure 3(c)). Cyclin B1 was about three and five times higher in VV-infected cells in comparison with nontreated cells, for 24 h and 48 h of infection, respectively. Such upregulation of cyclin B1 usually activates cell cycle arrest in the mitotic prometaphase.

In previous work, we observed that VV-GMCSF-Lact induced a higher percent of caspase-3 and caspase-7 activation, in time- and MOI-dependent manners, than VV-GMCSF-dGF [24]. In the current work, we revealed that the newly constructed VV-GMCSF-Apo produced the same amount of cells with active caspase-3 and caspase-7 as produced by VV-GMCSF-Lact (Figure 3(d)).

### 3.4. Mitochondrion Apoptosis

**Figure 4:**
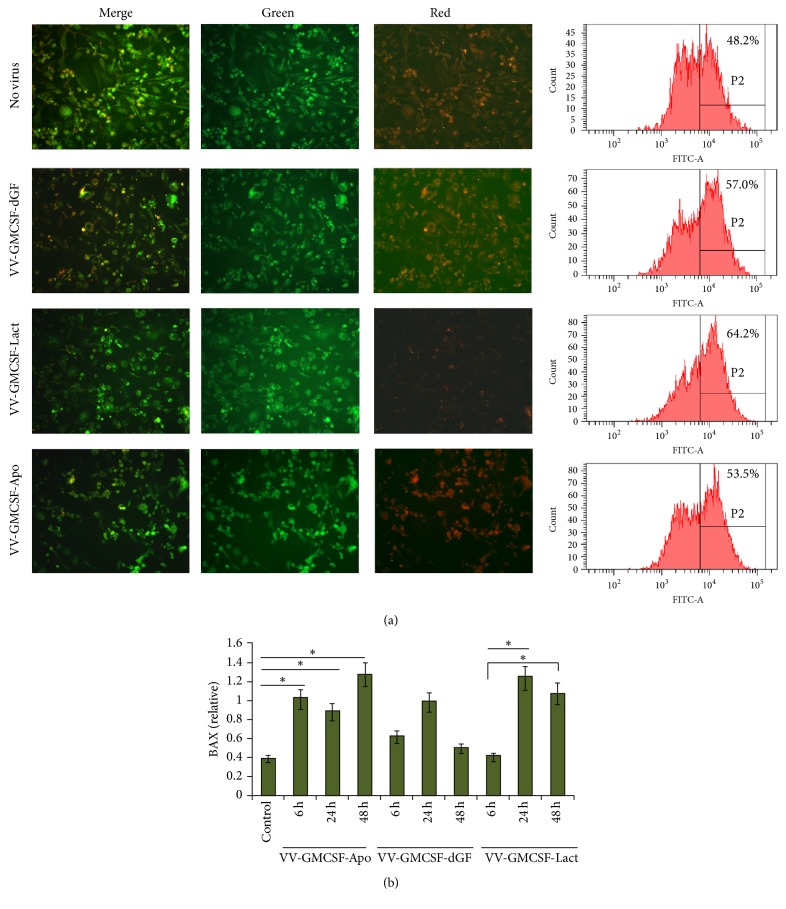
*Analysis of activation of mitochondrial apoptosis*. MDA-MB-231 cells were treated with VVs (0.05 PFU/cell). (a) Fluorescence microscopy and flow cytometry of infected cells (36 h after infection) treated with JC-1 indicator. Representative data of two independent experiments. (b) BAX expression in VV-infected cells (0.05 PFU/cell). Cells were treated with VVs for the indicated time. The quantification of western blot data was performed using Gel-Pro Analyzer software. Data are presented as mean ± SD (*n* = 2); ^*∗*^*p* < 0.05 versus control (nontreated cells).

Changes in mitochondrial membrane potential are also considered as an event of apoptosis. We used the membrane-permeable lipophilic cationic fluorochrome, JC-1, to detect virus-dependent transition of the mitochondrial membrane potential. The fluorescence emission shift detected by fluorescence microscopy or flow cytometry reflects the changes in mitochondrial membrane potential, since JC-1 monomers display green cytoplasmic fluorescence (*λ* = 525 nm), while its aggregates accumulated in functional mitochondria display orange fluorescence (*λ* = 590 nm) [31]. The changes in JC-1 mitochondrial binding are reflected by a decrease in the red/green fluorescence ratio. MDA-MB-231 cells and MX-7 cells were infected by recombinant VVs, and after 24 h of infection, cells were treated with JC-1 as described in Materials and Methods. Fluorescence microscopy data revealed the change in the red/green fluorescence ratio for cells treated with any of the three recombinant VVs in comparison with control nontreated human cancer cells, and mouse cancer cells (Figure 4(a), Figure S3). Flow cytometry analysis of the treated cells allowed us to estimate the relative JC-1 emission shift in the green channel, and we observed the highest increase of green fluorescence in cells treated with the lactaptin-coding recombinant VV-GMCSF-Lact (Figure 4(a), Figure S3).

According to the mitochondrion apoptosis pathway, activation of proapoptotic BH3 only proteins results in initiation and propagation of the effector caspases-3 and caspase-7. We analyzed BAX, one of the key BH3 only proteins, after VV infection. MDA-MB-231 cells were treated with recombinant VVs at various time points, and whole cell lysates were prepared for western blot analysis. We observed strong upregulation of BAX in virus-treated cells (Figure 4(b)). Treatments with recombinant viruses coding apoptosis-inducing proteins resulted in a tendency for a higher BAX increment than with the VV-GMCSF-dGF recombinant.

### 3.5. Features of Immunogenic Cell Death

**Figure 5:**
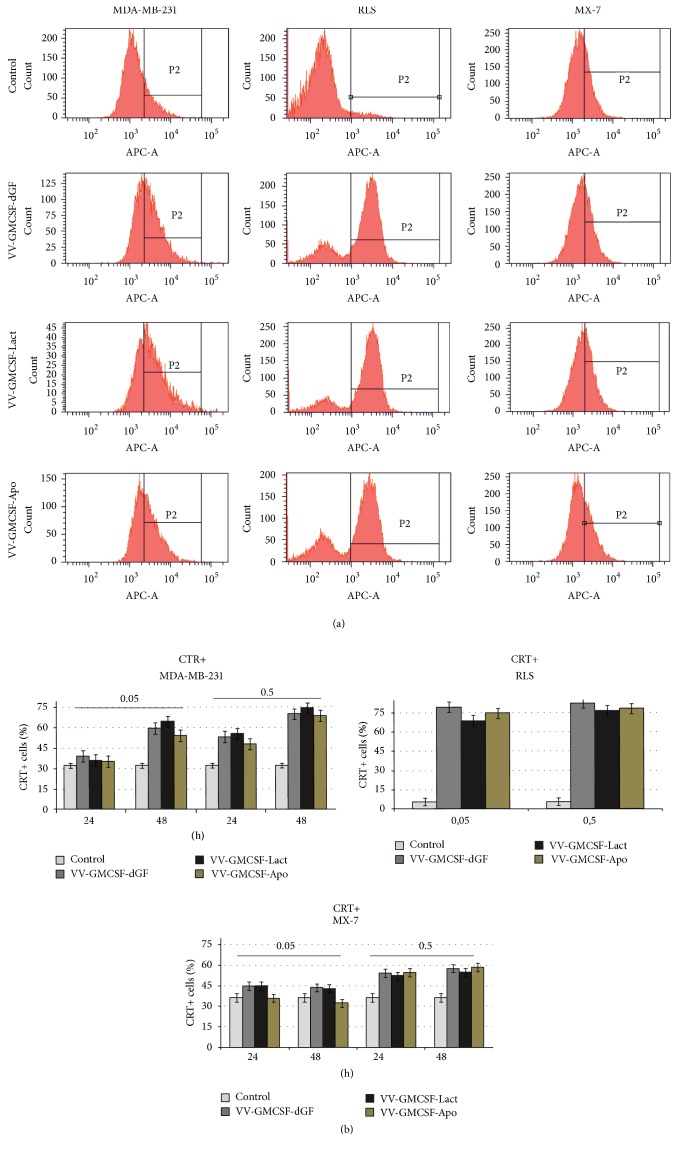
*Ecto-CRT analysis*. MDA-MB-231 and MX-7 cells were treated with VVs (0.05 and 0.5 PFU/cell) for the indicated time; RLS were treated for 24 h. Control cells were treated with PBS. Cells were stained with anti-CRT antibody, followed by immunofluorescence detection of CRT exposure. (a) CRT exposure after VVs treatment. Representative data of three independent experiments. Cells were treated with VVs (0.5 PFU/cell) for 24 h. Isotype control panel is presented for cells treated with PBS. (b) The results represent the % of CRT-positive cells ± SD.

Immunogenic cell death is characterized by the surface exposition of calreticulin and active release of ATP and HMGB1 into the extracellular environment [32]. To test ecto-CRT MDA-MB-231, MX-7 and RLS cells were treated with recombinant VVs; then nonfixed nonpermeabilized cells were stained using anti-CRT antibodies and analyzed by flow cytometry. Such sample preparation allowed us to determine that the visible increase in CRT-positive cells after virus infection resulted from the translocation of cellular CRT from endoplasmic reticulum to the outer cell membrane. We observed that recombinant VVs induced dose-dependent and time-dependent calreticulin exposure (Figure 5(a)). After 48 h of virus infection, the highest percentage of ecto-CRT-positive cells was observed for RLS mouse lymphosarcoma cells. CRT exposure was in concordance with the cytotoxicity for MX-7 cells: we did not observe significant differences in the relative amount of CRT-positive cells when compared to control cells or cells treated with low doses of recombinant vaccinia virus (0.05 PFU/cell) (Figure 5(b)). Comparing the ecto-CRT exposure induced by any of the three recombinant VVs, we found no differences in the efficiency of CRT translocation induced by recombinant viruses at the low dose as well as at the high virus dose for all tested cell lines.

**Figure 6:**
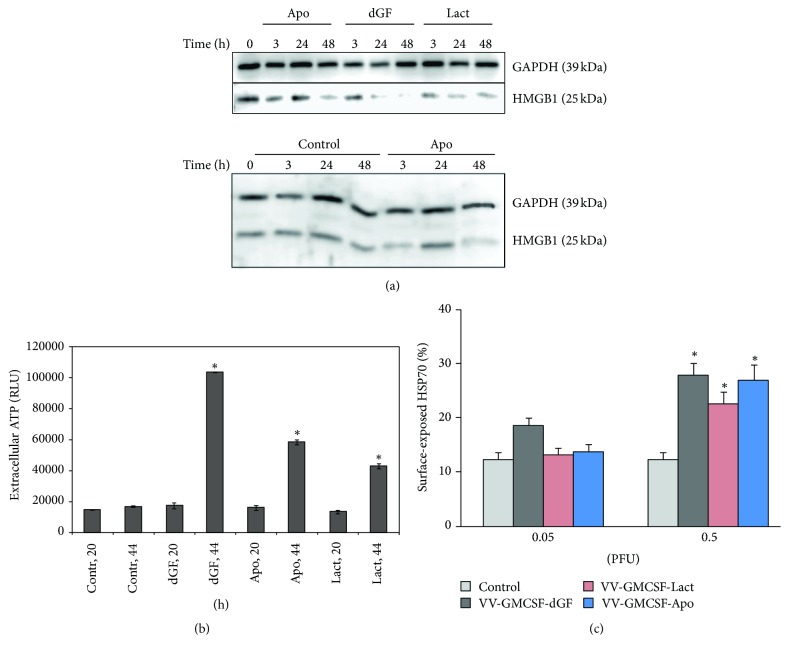
*Recombinant VVs induce immunogenic cell death in MDA-MB-231 cancer cells*. Control cells were treated with PBS. (a) Western blot analysis of HMGB1 expression in cell lysates 3, 24, and 48 h after infection (0.05 PFU/cell). One representative of three independent experiments is shown. (b) Relative amount of extracellular ATP, measured in conditioned media of virus-treated cells (0.05 PFU/cell). RLU: relative luminescent unit; (c) analysis of surface-exposed HSP70 by flow cytometry. Cells were stained 24 h after infection. ^*∗*^*p* < 0.05 versus control (nontreated cells).

Next, we checked changes of another ICD molecular determinant—HMGB1 protein. MDA-MB-231 cells were treated with recombinant VVs and intracellular HMGB1 was analyzed by western blot in lysate. We observed that cellular HMGB1 level in control samples was stable for different time points and VV treatment resulted in a decrease of cellular HMGB1 for all three recombinant VVs (Figure 6(a)).

No ATP release was observed after 20 h of virus infection, but after 44 h, the extracellular ATP was significantly higher compared to control for all three recombinant VVs (Figure 6(b)).

It was demonstrated that exposure of another DAMP component, the heat shock proteins HSP70 or HSP90, on the surface of dying cells is also an important component of ICD [33, 34]. In our experiments, we observed significant increments of surface-exposed HSP70 in the MDA-MB-231 cells after 24 h of VV infection (0.5 PFU/cell) (Figure 6(c)).

### 3.6. Virotherapy by Recombinant Vaccinia Viruses Coding Apoptosis-Inducing Proteins Delays Growth of Developed Tumor

**Figure 7:**
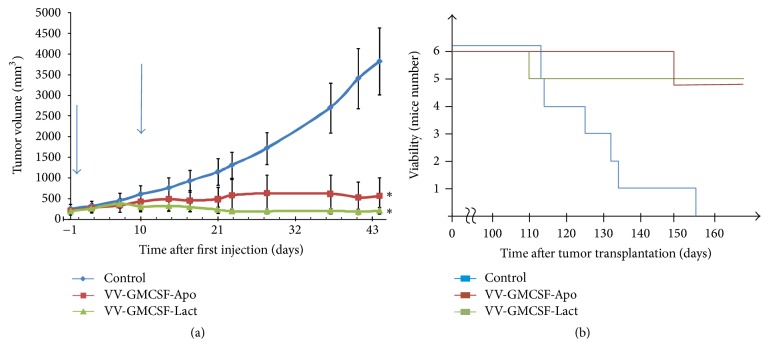
*Therapeutic effect of VV-GMCSF-Lact and VV-GMCSF-Apo injected into tumors*. (a) Established MDA-MB-231 tumors growing in SCID mice (*n* = 6 in groups) were injected locally with 1 × 10^7^ PFU of viruses followed by monitoring of tumor growth. Tumor growth curves are presented with SE. Arrows indicate the days of virus injections. Viability of treated mice is also represented (b); ^*∗*^statistically significant difference compared to the control (saline-treated) group (*p* < 0.003).

To analyze the efficacy of recombinant VVs coding apoptosis-inducing proteins in vivo against a large advanced tumor, MDA-MB-231 adenocarcinoma cells were introduced s.c. into SCID mice (day 0). After 42 and 51 days, mice received two intratumoral injections of VV (1 × 10^7^ PFU). Animals in the control group received intratumoral injections of saline. Median tumor volume in groups was estimated before the first mouse died (Figure 7(a)). A significant suppression in tumor growth was observed in mice treated with either VV-GMCSF-Lact or VV-GMCSF-Apo (94% and 85%, resp.). The investigation of survival rate in the experimental groups revealed a correspondence between tumor growth and survival: if the tumor was efficiently suppressed by recombinant viruses, then a high median viability was detected (Figure 7(b)). Virotherapy was well tolerated with no visible toxicity in mice.

## 4. Discussion

Over the past decade, many approaches based on oncolytic viruses have been tested. Great progress in cancer treatment has been made using attenuated oncolytic viruses coding various transgenes, as well as using a combination of oncolytic virus therapy and chemotherapeutic drugs or radiotherapy [7, 35–38]. Moreover, oncolytic viruses have efficiently killed cancer stem cells that were resistant to irradiation and chemotherapy [39].

By the new definition, cancer cell death induced by OVs is mostly immunogenic, though various OVs induce different types of cell death. For example, the main death route for cells infected by adenovirus is autophagy, and for vaccinia virus, the preferred mode of death is programmed necrosis [3, 5, 6]. In general, infection with vaccinia temporarily inhibits apoptosis to allow sufficient time for successful viral replication. VV mediates this apoptotic delay via upregulation of molecular pathways associated with expression of a set of antiapoptotic proteins, including serpins, the caspase inhibitors [40, 41]. Apoptosis suppression leads to necrosis or necroptosis of infected host cells [5]. We hypothesized that the expression of proapoptotic proteins, targeting different cell death pathways, can overbalance necrosis to apoptosis, resulting in achievement of higher tumor specificity and oncolytic activity. Lactaptin and apoptin, two proapoptotic proteins, were reported to efficiently induce apoptosis of various cultured cancer cells, via activation of different cell death pathways [26, 42, 43].

Previously, we reported that the attenuated double recombinant vaccinia virus, VV-GMCSF-Lact, coding the proapoptotic protein lactaptin and human GM-CSF, was a more powerful therapeutic for the treatment of drug-resistant lymphosarcoma [24]. Here, we have successfully engineered a new recombinant vaccinia virus, VV-GMCSF-Apo, that also expresses GM-CSF and the proapoptotic inducer apoptin. One of the main challenges for constructing recombinant VVs for anticancer therapy is obtaining strains that are strongly attenuated for replication in normal cells and characterized by a high replication rate in rapidly dividing cancer cells. This is possible due to inactivation of* thymidine kinase* and* virus growth factor* genes of VV. We demonstrated here that the double recombinant VV-GMCSF-Apo was 158 times more attenuated, and the double recombinant VV-GMCSF-Lact was 100 times more attenuated than the parental L-IVP strain of VV, ensuring tumor specificity of recombinant VVs.

The goal of the present study was to compare the features of cell death induction of these recombinant VVs in vitro and in vivo.

Some oncolytic viruses display aberrant, nonproductive infections in nonnative hosts, such as mice cells, leading to a mode of cancer cell death that differs with the native host [3]. Indeed, a comparison of oncolytic activity of double recombinant VVs in human and mouse tumor cells showed that MX-7 mouse rhabdomyosarcoma cells were less sensitive to both viruses, VV-GMCSF-Lact and VV-GMCSF-Apo, than MDA-MB-231 human adenocarcinoma cells. VV-GMCSF-Lact and VV-GMCSF-Apo were more potent in inducing cell death and apoptosis than recombinant VV coding GM-CSF alone in all tested cell lines.

The vaccinia genome encodes the Bcl-2-like protein, F1L, which helps to prevent apoptosis of host cells before virus replication by sequestering proapoptotic components [44]. F1L suppresses cytochrome c release by interacting with the proapoptotic Bcl-2 family members Bak and Bim [40, 45]. We have previously demonstrated that a recombinant analog of lactaptin induced the dissipation of the mitochondrial membrane potential and downregulation of the antiapoptotic Bcl-2 protein in treated MDA-MB-231 cells [42, 46]. Apoptin is also known as a regulator of the mitochondrial pathway of apoptosis, triggering cytochrome c release and caspase-9 activation in treated cells [47]. In the current work, we demonstrated that recombinant VV-GMCSF-Lact bearing the lactaptin transgene produced the highest changes in the mitochondrial membrane potential of infected cells, according to flow cytometry analysis and fluorescence microscopy. Taken together, these results suggest that lactaptin contributes to mitochondrial membrane destabilization leading to apoptosis. Moreover, both VV-GMCSF-Lact and VV-GMCSF-Apo induced upregulation of BAX expression, whereas VV-GMCSF-dGF induced a lower BAX increment. Our data indicate that expression of a proapoptotic stimulus can partly attenuate (directly or indirectly) viral antiapoptotic signals.

It is known that cyclin B1 is normally at or near peak level in metaphase, while it is low in G0. If cells are subconfluent, and external intervention is absent, then cyclin B1 is higher, relative to confluent cells [48]. We observed such characteristics in the present study when nontreated cells decreased the average expression of cyclin B1 growing to full confluency (data not shown). Conversely, we observed a linear increase of cyclin B1 during virus infection for all three recombinant VVs; thus recombinant VVs possibly induced cell cycle arrest in mitosis. A similar effect is typical for nocodazole-treated cells when long-term cell cycle arrest in mitotic prometaphase resulted in apoptosis [49]. Since no cyclin B1 differences were observed in cells treated with recombinant VVs, it is possible that expression of apoptosis-inducing proteins did not affect cyclin B1.

Activation of an endogenous antitumor immune response, resulting in immunological antitumor memory, is an attractive therapeutic strategy [50, 51]. Theoretically, tumor cells that die by ICD activate adaptive immune responses against tumor cell-specific antigens. Immunogenic cell death characteristics are mediated by damage-associated molecular patterns, which include the surface-exposed calreticulin and heat shock proteins, and release of adenosine triphosphate (ATP) and HMGB1 protein into the extracellular environment [19, 32, 52]. Typical features of ICD were demonstrated for cancer cells infected by an engineered adenovirus, coxsackievirus B3, Newcastle disease virus, and others [20, 53, 54]. We investigated here whether the expression of different transgenes influences the ability of recombinant VVs to induce ICD in MDA-MB-231 adenocarcinoma cells. This is important because the expression of transgenes can sometimes inhibit the potency of viruses [55]. We demonstrated in vitro that recombinant VVs caused calreticulin and HSP70 exposure, decreased cellular HMGB1, and increased extracellular ATP. According to the engineering scheme, the tested double recombinant VVs differ from each other by* vgf* gene insertions, but all code the GM-CSF protein. GM-CSF can stimulate transformation of monocytes to macrophages that are able to execute antigene-presenting function. The lack of significant differences in ICD markers in VV-infected cells likely indicates the importance of GM-CSF expression as well as VV properties, but not the expression of proapoptotic proteins for VV-induced ICD. Moreover, there are data showing that an additional immunomodulator gene deletion in VV increased attenuation but decreased immunogenicity, compared with a single gene deletion [56].

Here we have demonstrated a strong decrease in cellular HMGB1 in comparison with nontreated cells, but an estimation of extracellular HMGB1 was not performed. The strong decrease of cellular HMGB1 is in agreement with literature showing that cancer cells secrete and overexpress HMGB1 by stimulation of growth factors, cytokines, and cellular stress [57]. Regarding the lack of increment of intracellular HMGB1 for all three VVs, this suggests that the decrease was due to and in proportion with HMGB1 release.

It is now clear that the response of tumor-bearing mice to treatment with recombinant vaccinia viruses is complex. We have previously demonstrated that VV-GMCSF-Lact was more efficient in delaying growth of MDA-MB-231 cells in SCID mice than VV-GMCSF-dGF [24]. Here, we reveal VV potential against large tumor nodes: before the beginning of OV therapy, tumors reached an average size of 7 × 7 × 7 mm and had good vascularity. We observed that both double recombinant VVs, coding apoptosis-inducing proteins, blocked tumor growth very efficiently. Only two injections of recombinant VVs led to tumor growth inhibition of up to 94% for VV-GMCSF-Lact and 85% for VV-GMCSF-Apo. It would be noted that lactaptin is not the only human milk protein to be cloned into oncolytic virus genome for improving its antitumor activity. Wang et al. [58] demonstrated that the adenoviral vectors carrying human lactoferrin significantly inhibited tumor growth with apoptosis-related morphological changes in tumor nodes. Thus, the engineering of recombinant oncolytic viruses containing bioactive milk proteins might be a promising strategy for anticancer therapy.

## 5. Conclusions

Overall, our results demonstrate that double recombinant VVs coding GM-CSF and the proapoptosis proteins, lactaptin and apoptin, differ from the recombinant VV coding only GM-CSF in the induction of apoptosis in cancer cells, but not in the induction of ICD.

## Supplementary Material

S1 Fig. Visualization of lytic (apoptotic) morphology of infected MX-7 cells. Cells were infected with VV-GMCSF-dGF, VV-GMCSF-Lact and VV-GMCSF-Apo. After 24 and 48 h post-infection, cells were analyzed by inverted microscope (phase-contrast) and photographed. Representative photomicrographs of three independent experiments. S2 Fig. Visualization of lytic (apoptotic) morphology of infected MCF-7 cells. Cells were infected with VV-GMCSF-dGF, VV-GMCSF-Lact and VV-GMCSF-Apo. After 6, 24 and 48 h post-infection, cells were analyzed by inverted microscope (phase-contrast) and photographed. Representative photomicrographs of three independent experiments. S3 Fig. Mitochondrial apoptosis activation in MX-7 cells. Cells were treated with VVs (0.05 PFU/cell). (A) Fluorescence microscopy of infected cells treated with JC-1 indicator. (B) Flow cytometry analysis of VV-infected cells stained with JC-1.

## Figures and Tables

**Table 1:** Virulence of VVs.

Virulence	VV-GMCSF-Lact	VV-GMCSF-Apo	L-IVP
^*∗*^LD_50_	10^3.9^	10^3.7^	10^5.9^
Attenuation^*∗∗*^	100 (10^2^)	158 (10^2.2^)	—

^*∗*^LD_50_ expressed as a tenfold dilution of VVs starting from 10^−1^ (dose 10^7^ PFU/egg) causing 50% mortality of embryos. ^*∗∗*^Attenuation was calculated as the difference of decimal logarithms of LD_50_ of recombinant and parental L-IVP strains.
